# Optimized protocol for detection of native, full‐length HIV‐1 envelope on the surface of transfected cells

**DOI:** 10.1002/hsr2.74

**Published:** 2018-07-31

**Authors:** J.B. Altman, X. Liu, V. Itri, S. Zolla‐Pazner, R.L.R. Powell

**Affiliations:** ^1^ Division of Infectious Diseases, Department of Medicine Icahn School of Medicine at Mount Sinai New York NY USA

**Keywords:** Env, expression, HIV, transfection

## Abstract

**Aims:**

Designing therapeutics against the HIV envelope glycoprotein (Env) is only as accurate as the structure of the Env they are targeting. Conserving the structure of the Env trimer is crucial for proper experimental assessment of antibody binding and neutralization. However, Env is notably difficult to express by transfection of a recombinant Env plasmid. To increase surface expression, researchers commonly utilize c‐tail mutants of the gp41 transmembrane glycoprotein of HIV‐1, but mutations and deletions in this region can impact the overall conformation and stability of the Env trimer. Multiple studies have shown that while tail mutants have higher Env surface expression, they are easier to neutralize and have altered trimer conformations compared with wild‐type Env found in vivo on infected cells. To assess and characterize native cell surface Env structures, we sought a protocol that could reliably detect wild‐type Env surface expression by flow cytometry.

**Methods and results:**

By avoiding fetal bovine serum–based buffers, significantly increasing the amounts of transfected plasmid and Env‐specific antibody and by selecting a bright, biotin + streptavidin‐PE detection system, we were able to increase the surface expression of transfected Env protein.

**Conclusion:**

This protocol will allow for more precise assessment of antibody binding, epitope exposure, and Env structure, all of which will contribute to designing more effective vaccines and immunotherapeutics.

## 1 INTRODUCTION

In order to study HIV entry into cells, it is necessary to evaluate the virus' glycoprotein, envelope (Env). Without a functional Env, the virus cannot enter the host cell, making Env an attractive target for therapeutics.^1^ Studying the glycoprotein in an in vitro setting entails transfecting plasmids containing Env into cells. However, few proteins end up getting expressed on the cell surface, and without a sufficient number of glycoproteins, interactions can be hard to measure.^2^ To increase the density of cell surface Env, researchers have utilized Env that lacks the C terminal tail (c‐tail). This mutation increases fusion activity, viral entry, syncytia formation, and Env surface expression.^3^, ^4^, ^5^, ^6^ C‐tail mutants increase Env surface expression by approximately eightfold in 293T cells, inducing similar increases in virus neutralization sensitivity and Env antibody (Ab) titers elicited in animal studies.^7^, ^8^, ^9^ These mutants elicit greater Ab diversity than wild‐type Env, likely because of conformational changes that expose more antigenic sites.^9^, ^10^ These conformational changes do not represent native Env protein, and it is unlikely virions in vivo would survive with these newly exposed antigenic sites. Therefore, using c‐tail Env to study Env structure or Env‐receptor interaction, or to design Env targeted therapeutics, is not ideal. Furthermore, studies have shown the dependence of increased Env expression to elicit an antibody response against the glycoprotein.^11^ Increasing Env surface expression should not greatly impact the virus, as the number of Envs that can be incorporated into viral particles is limited; this includes those produced by 293T cells (used to generate pseudovirions). This is because the virion has a minimal Gag‐to‐Env ratio^12^ of 45:1 to 70:1. Thus, increasing Env surface expression should not have any deleterious effects on pseudovirus production. While previous studies have used WT Env to study cell‐cell infection and produce pseudovirus, we have found that using conventional amounts of WT Env (as described for pseudovirus production in Li et al^13^ to study antibody‐Env interaction) is simply not sufficient, as there is not enough Env signal to detect antibody‐Env interaction by flow cytometry. Additionally, detection of Env on the cell surface has proven tricky. Studying the distribution of viral proteins at small spatial scales requires an optical resolution that is beyond the limit of light microscopy (∼200 nm) and is only visible with new super‐resolution fluorescence microscopy techniques.^14^ Fluorescence microscopy of HIV‐1–producing cells shows patchy signals of both Gag and Env at the plasma membrane with low correlation coefficients, and the resolution of light microscopy is not sufficient to discern adjacent individual budding sites.^14^ Therefore, increasing native Env expression will aid not only in studying antibody responses to Env but also in visualizing it and its interactions as well. Since most studies of surface expression have used c‐tail mutants, and expression of native Env is typically poor, we sought a more sensitive protocol for expressing and detecting native cell‐surface Env for future structural studies and analyses of Ab binding.

## 2 MATERIALS

### 2.1 Cells

For all experiments, 293T cells (ATCC #CRL‐3216) were grown in vented T‐75 flasks using complete 10% fetal bovine serum (FBS) DMEM media (Life Technologies catalog #11965092). Cells were obtained from and authenticated by ATCC using morphology, karyotyping, and PCR‐based methods. Cells were used for the experiments described herein, within 6 months of resurrection. Cells were split approximately twice per week. For transfections, 3 × 10^6^ cells were seeded in T‐75 flasks with 12 mL of complete media and left to grow overnight, followed by transfection the following morning.

### 2.2 JRFL envelope expression plasmid

pCAGGS_JR‐FL.JB gp160 (originally supplied by John Mascola) was used for these experiments.^15^ This plasmid encodes the entire gp160 region of the HIV‐1 envelope (6225‐8795 locus in HXB2 numbering; accession number AAB05604). pCAGGS vector information is available at https://www.addgene.org/vector-database/2042.

### 2.3 Antibodies

Monoclonal antibodies (mAbs) were isolated from PBMCs of HIV‐1–infected individuals as described previously.^16^ All mAbs (CD4 targeted: 559^17^; 3‐targeted: 2424^18^; V2‐targeted: PG9,^19^ 830A,^20^ 1357,^21^ 697‐D,^22^ 2158^23^; gp41‐targeted: 98‐6,^24^ 4e10,^25^ 240‐D^26^; and negative controls: 3685^27^ [anthrax], 1418^28^ [parvovirus]) were generated in‐house with the exception of PG9, which was obtained from the AIDS Reagent Repository (catalog #12149).^16^

### 2.4 Flow cytometry

Cells were assessed with a BD Fortessa flow cytometer, and 2000 events were collected in the phycoerythrin‐positive (PE+) gate. 293T cells were selected from a plot of forward v. side scatter (FSC/SSC) from which doublets were excluded in a forward scatter height vs forward scatter area plot (FSC‐H/FSC‐A). Live cells were selected by gating, and PE‐positive cells in this gate, representing Env‐stained cells, were quantified as positive in a PE histogram by comparing with cells in which no primary mAb was used. Normalized “Env scores” were calculated as the number of cells in the PE‐positive gate multiplied by the geometric mean MFI of that gate,^6^ divided by 10.

## 3 METHODS

The detailed protocol used is described below:

1. Seed 3 million cells in 12 mL of complete DMEM. Incubate overnight.
2. Transfect with 20 ug of pCAGGS_JR‐FL.JB gp160
  - Based on the volume of Env needed for 20 μg, add DMEM (no FBS) up to 100 μL in a 1.5‐mL tube, and mix. In another tube, add 652‐μL DMEM (no FBS) and 48 μL of FuGene 6 (Promega catalog #E2691) (pipet the FuGene directly into the media, do not touch the tube), and mix.
  - Transfer the tube containing JRFL Env into the FuGene tube, and mix well.
  - Incubate at room temperature (RT) for 30 minutes.
  - Add to the media in 293T flask. Swirl the media around the flask and incubate for 5 hours at 37°.
  - Remove the media, and carefully add 15‐mL fresh complete DMEM.
  - Incubate overnight.
3. Harvest the cells the next day (24 hours from transfection)
4. Decant the media and wash the flask 5 mL of PBS (be careful not to detach the cells).
5. Incubate with 5 mL gentle cell dissociation buffer (about 3 min) until the cells are detached (Life Technologies catalog #13151‐014).
6. Add 5 mL complete DMEM, and pipet up and down to remove all cells from the flask. Transfer to a 50 mL conical tube and spin down for 5 min at 1200 rpm.
7. Decant the supernatant, add 10 mL of PBS, and spin again.
8. Decant the supernatant. Resuspend the cells in 5 mL of PBS, and take an aliquot for counting. Spin again.
9. Resuspend cells at 1 million/mL.
10. Stain with 1 μL/mL of viability dye (BD catalog #562247) in the dark at 4°C for 30 minutes. Spin again.
11. Decant the supernatant. Resuspend the cells in 5 mL of PBS. Spin again.
12. Repeat step 11.
13. Block the cells with 3% BSA (Gibco catalog # 15260037) at 4°C for 1 hour. Vortex every 20 minutes.
14. During the block, seed a 96‐well plate with your primary antibodies starting at 200 μg/mL in 200 μL, and perform twofold dilutions down to 3.125 μg/mL. Place in fridge.
15. Spin cells. Decant the supernatant. Resuspend the cells in 5 mL of 1% BSA. Repeat 2×.
16. Count cells, and resuspend in 1% BSA to 500 000 cells/mL.
17. Add 100‐μL cells to each well of antibody plate and mix. Incubate in fridge for 30 minutes.
18. Spin down the plate at 2000 × *g* for 10 minutes at 4°. Wash with 100 μL 1% BSA. Perform 2 washes.
19. Add 100 μL 1:500 Biotin IgG (Abcam catalog #ab97223) to each well. Incubate in fridge for 30 minutes. Repeat step 18.
20. Add 100 μL 1:1000 streptavidin PE (Biolegend catalog #405204) to each well. Incubate in fridge for 30 minutes. Repeat step 18.
21. Add 200 μL 0.5% formaldehyde to each well. Place in fridge until analysis.

## 4 RESULTS

Initially, we aimed to determine baseline detectable Env expression levels. Four micrograms of Env JR‐FL plasmid was used to transfect 293T cells, which were then incubated at 37°C after media replacement for 24 or 48 hours. Transfections were performed using FugeneHD transfection reagent according to the manufacturer's protocol (Promega catalog #E2311) with the reagent being left on the cells in media for 5 hours before media replacement, based on the standard method for generating HIV pseudovirus from 293T cells.^29^ Cells were detached with gentle dissociation buffer (Life Technologies catalog #13151‐014) according to the manufacturer's protocol and washed 3 times in phosphate‐buffered saline (PBS pH 7.4; Life Technologies catalog #10010023). Cells were incubated for 30 minutes at 4°C with a V3‐specific human (mAb 2424) and with a parvovirus‐specific control human Ab (1418) at a starting concentration of 50 μg/mL, which was titrated fourfold. Primary antibodies were incubated for 30 minutes, followed by 2 washes and incubation with antihuman IgG‐Allophycocyanin (APC) (BD) at a standard 1:1000 dilution at 4°C for 30 minutes in the dark. Antibody staining and washes were performed in FACS buffer (PBS + 2.5% FBS). Substantial cell death was seen with the 48‐hour transfection, and neither the 24‐ nor 48‐hour transfection yielded detection above that of the mock‐transfected control (data not shown). It was clear that the background needed to be reduced to detect Env.

**Figure 1:**
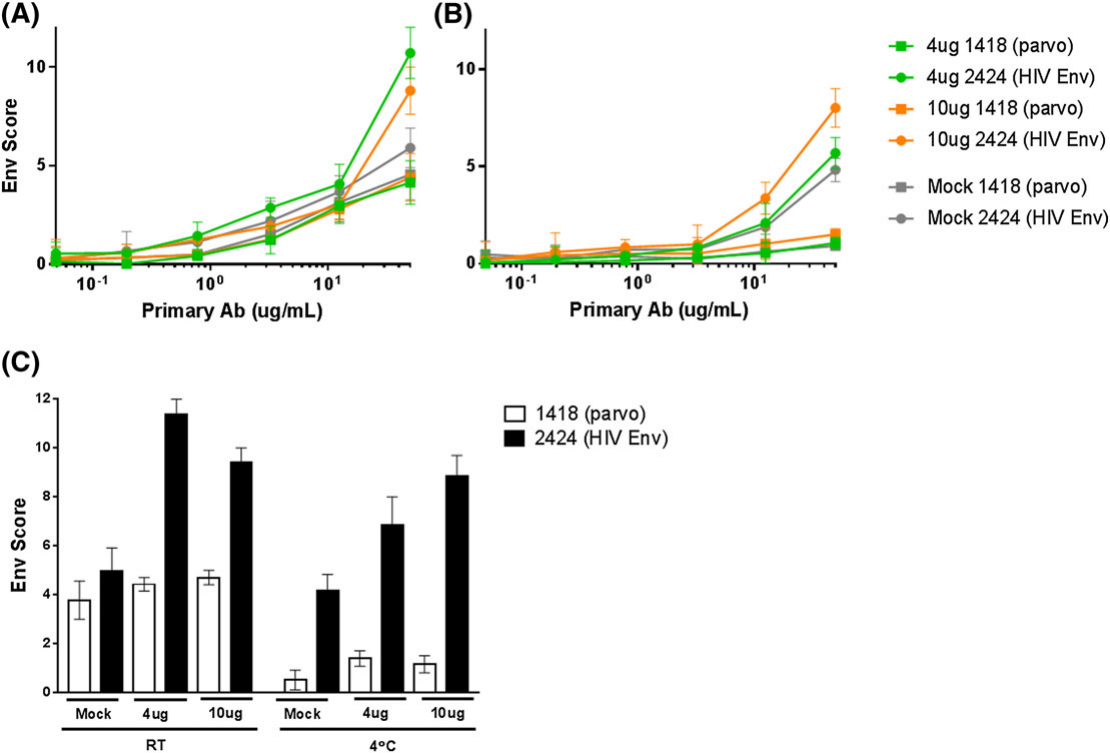
4°C staining improves Env detection. 293T cells were mock‐transfected or transfected with 4 or 10 μg of Env plasmid as described in Section 3. The Env‐V3 mAb 2424 was used to detect transfected Env on the cell surface, and the Parvovirus mAb 1418 served as negative control. MAbs were titrated down from 50 μg/mL. Staining was performed at room temperature (RT) (A) or 4°C (B). (C) 50‐μg/mL mAb titration curve values demonstrate reduced 1418 staining at 4°C compared with RT, as well as improved 2424 signal at 10‐μg transfection at 4°C compared with the mock‐transfected cell. Experiments were repeated 3 times. Mean values and SEM are shown

In order to reduce nonspecific binding, 1% BSA was used in place of FBS for all buffers, and a 1‐hour 3% BSA blocking step was included after the viability stain in an effort to further reduce background signal. To better amplify the Env signal, staining was switched from antihuman IgG‐APC to antihuman Biotin IgG (Abcam catalog #ab97223) + streptavidin‐PE (Biolegend catalog #405204), both at 1:1000 dilution. As expected, PE resulted in a log‐increased signal range. Under these conditions, we compared RT and 4°C staining of mock, 4‐, and 10‐μg transfections (Figure 1A‐C). At RT, the 4‐μg transfection yielded ~20% higher 2424 Env detection (Env score 10.7) compared with the 10‐μg transfection (Env score 8.8), which was at least twofold higher than that detected for the mock transfection (Figure 1A). Nonspecific binding of 1418 was similar for both amounts of transfected plasmid at RT and for the mock‐transfected control, indicating Env transfection was not increasing 1418 binding to the cell surface, but rather, this mAb exhibited an inherent binding to the cells. At 4°C, the 1418 background binding levels were greatly reduced, while the 2424 binding to the mock transfection remained similar to the RT experiment, and the 10‐μg transfection exhibited the best 2424 Env detection (Env score 8), which was ~30% higher than that for the 4‐μg transfection (Figure 1B). Although 2424 binding levels to control cells remained high, we chose to proceed with the 4°C 10‐μg conditions because of the lowered 1418 staining and increased 2424 Env detection.

**Figure 2:**
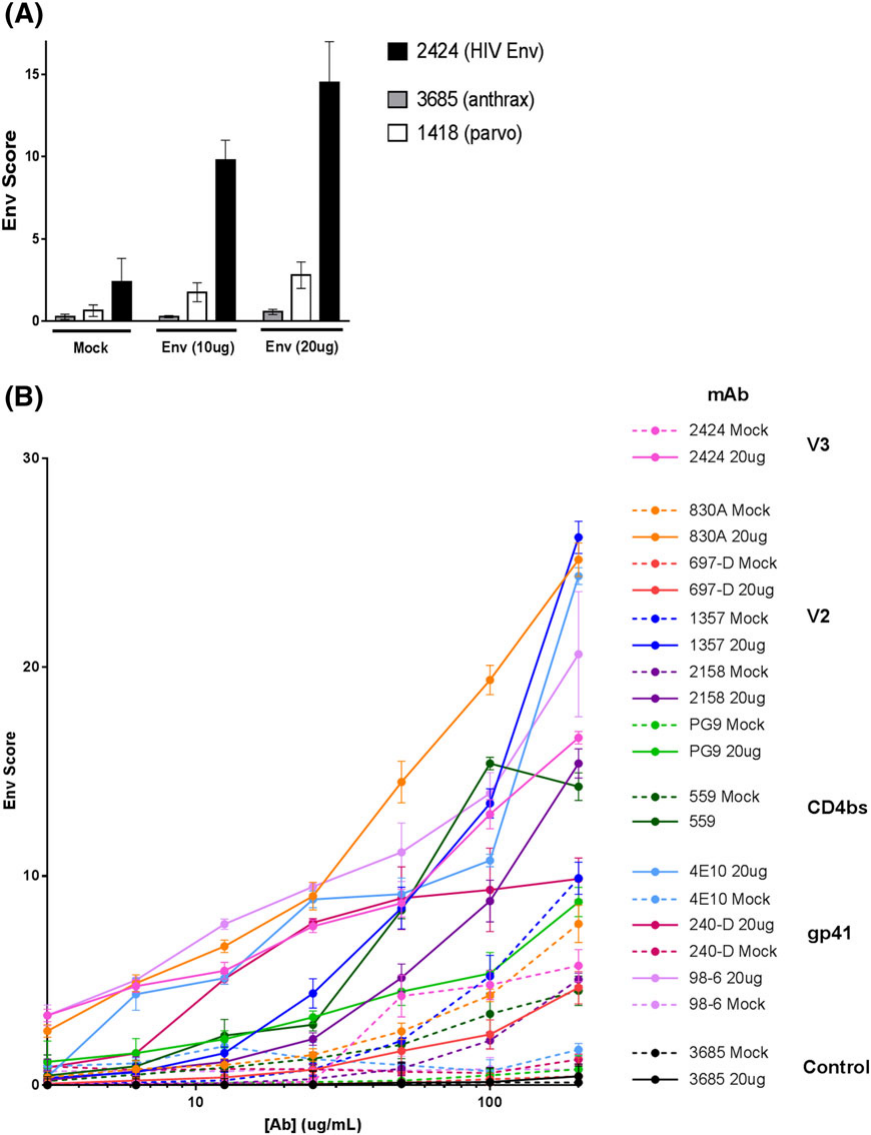
Atypically high concentrations of transfected plasmid and anti‐Env mAbs allows for improved detection of native HIV Env. A, Cells were mock‐transfected or transfected with 10 or 20 μg of Env plasmid as above and stained with 50 μg/mL of the mAb indicated. The Anthrax mAb 3865 was assayed and found to be a true negative control compared with the 1418 parvovirus mAb. B, Detection of native Env by various V3‐, V2‐, CD4 binding site‐ and gp41‐specific mAbs. Titration curves starting at a mAb concentration of 200 μg/mL to stain 20 μg‐Env‐transfected and mock‐transfected cells are shown. Experiments were repeated 3 times. Mean values and SEM are shown

We proceeded to test a 20‐μg transfection as, at 4°C, it appeared that increased plasmid may improve Env detection. Given 1418 exhibited inherent binding to 293T cells, we tested mAb 3865, specific for an Anthrax surface protein. The Anthrax mAb exhibited virtually no binding to mock‐ or Env‐transfected 293T cells (Figure 2A). The 20‐μg transfection yielded a ~45% signal increase for 2424 compared with 10 μg, with a very minimal increase in control mAb signal. A 40‐μg plasmid transfection was tested but led to widespread cell death (data not shown). It was notable from the titration curves (Figure 2B**)** that even at 50‐μg/mL mAb, saturation of the Env signal was not observed, nor was there a prozone/“hook effect,” wherein too much mAb can inhibit binding and give the same result as not enough mAb in the assay.^30^ We hypothesized that due to the minimal amount of Env on the cells' surface, an increased mAb concentration might further improve Env detection. MAbs were titrated from 200 μg/mL (Figure 2B). At these conditions, additional Env mAbs were tested, including the V2‐specific mAbs 830A, 697‐D, 2158, and PG9; the CD4 binding site‐specific mAb 559; and gp41 MPER mAb 4e10, gp41 cluster II mAb 98‐6, and the gp41 cluster I mAb 240‐D (Figure 1E). Even at 200 μg/mL, all but 559 did not reach saturating Env detection, and for all mAbs, Env signal under these conditions was at least twofold to threefold above the mock‐transfected controls.

## 5 DISCUSSION

The ability to study HIV Env in order to better understand virus‐receptor interaction and design therapeutics is contingent upon being able to recreate WT native Env protein. Because of the inability to express Env in sufficient quantity on the cell surface, previous investigators have utilized tail mutants.^31^ This method succeeds in placing more Env on the membrane, but at the cost of structural and antigenic accuracy.^8^, ^9^ Without the proper native structure and antigenic sites, there is no guarantee that therapies designed in these systems will be effective in vivo. Here, we describe how to express and detect native Env without utilizing a c‐tail mutant. While tail truncated Env plasmids result in increased Env surface expression, numerous studies have highlighted the important conformational differences between c‐tail mutated and wild‐type Env,^3^, ^4^, ^7^, ^9^ necessitating a method to study cell‐surface Env in its native conformation. Through a series of experiments aimed at optimizing Env cell surface expression, we found that performing all of the Ab incubations at 4°C helped to reduce nonspecific binding, as did eliminating all FBS from the assay buffers. Additionally, increasing the amount of JR‐FL Env plasmid that was transfected (20 μg) into the 293T cells resulted in a marked increase in Env surface expression, and use of an unusually high amount of primary Ab, as well as selection of a true negative control Ab, allowed for highly improved Env detection. With these improvements, we were able to readily detect native Env on the cell surface by flow cytometry, at a level significantly above background. As Env expression is low on infected cells and virions—in an effort to evade immune detection—increased expression might not be ideal for certain assays. Increasing Env expression in an in vivo setting might trigger the immune system more quickly and severely than WT virus. However, as we are using this in vitro to assay for antibody binding, this is not a concern in our system. Additionally, effector function assays could be augmented in the presence of increased Env density. However, killing assays could be performed, as increased Env expression could enhance effector function and allow for detection/study of MHC and antibody independent lysis.^32^ As we have demonstrated, flow cytometry is able to quantify antibody binding better with increased cell surface Env expression. Similarly, microscopy‐based experiments would be better able to visualize Env, as lowly expressed proteins/receptors are hard to detect.^14^ Cell to cell infection/fusion might also increase as a result of increased Env, as virological synapses form between CD4 and Env.^33^ The last 5 years have exposed new roles for Env, including its recruitment to the surroundings of Gag assembly sites, dependent on the presence of its cytoplasmic domain.^14^ As we learn more about Env and the multiple roles it plays besides entry in HIV infection, the more crucial it is to be able to study this glycoprotein in its native form. Expressing native Env on the surface of 293T cells will allow for more precise assessments of Ab binding, epitope exposure, and Env structure, all of which will contribute to designing more effective vaccines and immunotherapeutics.

## AUTHOR CONTRIBUTIONS

Conceptualization: Susan Zolla‐Pazner, Rebecca Powell

Formal analysis: Rebecca Powell, Jennie Altman

Funding acquisition: Susan Zolla‐Pazner

Investigation: Rebecca Powell, Jennie Altman

Methodology: Susan Zolla‐Pazner, Rebecca Powell, Jennie Altman

Project administration: Rebecca Powell

Resources: Vincenza Itri, Xiaome Liu

Supervision: Susan Zolla‐Pazner, Rebecca Powell

Validation: Rebecca Powell, Jennie Altman

Visualization: Rebecca Powell, Jennie Altman

Writing—original draft preparation: Jennie Altman

Writing—review and editing: Rebecca Powell, Susan Zolla‐Pazner, Jennie Altman

## REPRINT REQUESTS

Rebecca Powell, PhD, Assistant Professor, Division of Infectious Diseases, Icahn School of Medicine at Mount Sinai, One Gustave L. Levy Place, Annenberg 23‐50, New York, NY 10029, USA.Email: http://rebecca.powell@mssm.edu
